# Mer Tyrosine Kinase (MERTK) modulates liver fibrosis progression and hepatocellular carcinoma development

**DOI:** 10.3389/fimmu.2022.926236

**Published:** 2022-08-08

**Authors:** Rosaria Maria Pipitone, Vincenza Calvaruso, Lorenza Di Marco, Francesca Di Salvo, Miriam Gaggianesi, Giulia Lupo, Rossella Zito, Claudia La Mantia, Matteo Ramazzotti, Salvatore Petta, Vito Di Marco, Antonio Craxì, Stefania Grimaudo

**Affiliations:** ^1^ Department of Health Promotion, Mother and Child Care, Internal Medicine and Medical Specialties, University of Palermo, Palermo, Italy; ^2^ Gastroenterology Unit, Department of Medical Specialties, University of Modena e Reggio Emilia, Modena, Italy; ^3^ Department of Agricultural, Food and Forest Sciences, University of Palermo, Palermo, Italy; ^4^ Department of Surgical Oncological and Stomatological Sciences, University of Palermo, Palermo, Italy; ^5^ Department of Biochemical, Experimental and Clinical Sciences, University of Florence, Florence, Italy

**Keywords:** Mer Tyrosine Kinase polymorphism (MERTK polymorphism), liver fibrosis, hepatocellular carcinoma, matrix metallopeptidase, WNT gene family pathway (WNT pathway)

## Abstract

MerTK is a tyrosine kinase receptor that belongs to the TAM (Tyro3/Axl/Mer) receptor family. It is involved in different processes including cellular proliferation/survival, cellular adhesion/migration, and release of the inflammatory/anti-inflammatory cytokines. Although it is reported that MERTK polymorphisms affect the severity of viral and metabolic liver diseases, being able to influence fibrosis progression and hepatocellular carcinoma development, the mechanisms remain unknown. Methods: using a microarray approach, we evaluated the liver expression of genes involved in fibrogenesis and hepatocarcinogenesis in patient with chronic hepatitis C (CHC), stratified for MERTK genotype and MERTK expression. Results: we found that the rs 4374383 AA homozygosity is associated with lower MERTK expression in CHC patients and that, depending on MERTK genotype, Matrix Metallopeptidase 9 (MMP9), Matrix Metallopeptidase 7 (MMP7), Secreted Frizzled Related Protein 1 (SFRP1) and WNT gene family 11(WNT11) show differential expression in patients with CHC with or without neoplastic progression. Conclusions: our results confirm that MERTK represents a genetic biomarker for progression of liver disease and are suggestive of translational relevance for the study of downstream pathways involved in fibrogenesis and hepatocarcinogenesis.

## Introduction

Hepatitis C Virus (HCV) infection is a major etiology of hepatocellular carcinoma (HCC) worldwide. Even in the direct-acting antivirals era, HCV-related HCC occurrence remains significant (1). In this context, the role of genetic background in conditioning HCV-induced liver disease progression is crucial and host genetics should be considered in HCC surveillance to identify those patients who need more rigorous monitoring.

Mer tyrosine kinase (MerTK) is a major macrophage receptor involved in the clearance of apoptotic cells and it is expressed mainly in subsets of alternatively activated (M2) macrophages (2). Tumor-associated macrophages mostly display an M2-like phenotype; they are major sources of cytokines and participate in tumor progression, angiogenesis, and tissue remodeling (3, 4).

MerTK is involved mainly in efferocytosis, but it can also induce the fibrogenesis acting directly on the hepatic stellate cells (HSCs) (5, 6). Genome Wide Association Study (GWAS) reported that a Single Nucleotide Polymorphism (SNP) of MERTK (rs4374383 A>G) is associated with the risk of developing fibrosis in patients with Chronic Hepatitis C (CHC) (7). It seems that the polymorphic status of MERTK gene may play a key role not only in the control of apoptosis, in the epithelial-mesenchymal transition of HSCs and in the immune response, but also in the mechanisms involved in fibrosis progression and carcinogenesis (8).

Starting from this evidence, we investigated whether the MERTK variant A>G can influence the risk of progression of liver disease, in patients with chronic infection sustained by hepatitis viruses (C or B) or with nonalcoholic steatohepatitis (NASH).

Preliminary data showed that the AA MERTK genotype can significantly affect the risk of liver decompensation (LD) and HCC development in patients with HCV cirrhosis, treated with Peg-interferon alfa-2b and ribavirin (9).

On the other hand, the association between the presence of A allele of MERTK and the risk of HCC development was observed in patients with chronic Hepatitis B Virus (HBV) infection with active HBV replication or virological suppression. By evaluating the incidence of HCC according to the disease stage and the genotype, the probability of HCC development was higher in patients with liver compensation (LC) and AA/GA genotype compared to patients with LC and GG genotype. In addition, the incidence of HCC in Chronic Hepatitis B (CHB) patients without cirrhosis and with GA/AA or GG genotype was lower and similar, respectively, compared to those with cirrhosis (10).

These results confirm that the association between MERTK polymorphism and HCC is virus-independent, being observed both in HCV and HBV infections, and correlates with the inflammatory response and probably with the mechanisms of angiogenesis and tumorigenesis.

In this perspective the polymorphic status of MERTK gene represents a genetic condition able to influence the progression of liver disease: on one hand, AA/GA genotype of rs4374383 is protective against fibrosis progression according to Patin, on the other, the A allele confers a significant additional risk for HCC development. It has been reported that AA genotype, which is associated with lower intrahepatic expression of MERTK, is protective against severe fibrosis also in Non-Alcoholic Fatty Liver Disease (NAFLD) through a mechanism involving the modulation of HCS activation (11).

However, the rs4374383 (A>G at chr2:112013193) SNP of MERTK gene does not fall within regulatory regions and locates in the 8thintron. The aim of the present paper is to investigate the molecular mechanism involved in both protection from liver fibrosis and HCC development in CHC patients with AA genotype.

The rs4374383 is in string Linkage Disequilibrium (LD) with rs6726639 (A>C at chr2:111995520), localized at the beginning of the 4thintron on MERTK gene (r2 = 0.94, 17673 bp distance). The LD between the two variants is very strong in the 1000 genomes project (12, 13)

Actually, the rs4374383 and rs6726639 variants holds many other variants at very high LD (i.e. r^2^ ± 0.9). In Italy (Tuscany in Italy dataset), at least 78 other variants are shared. The GTEx database indicate that the expression of MERTK in liver is mainly due to isoforms 5 and 6. Accordingly, it makes no sense to investigate rs4374383 and rs6726639 separately because their LD is so high that their results will always be the same.

The GTEx portal contains per-tissue/organ differential quantitative RNA data (termed expression quantitative trait loci, eQTLs), i.e. genes that have differential expression in the presence of a variant. GTEx indicates that the two variants rs4374383 and rs6726639 (reasonably the entire LD region) in liver are associate with altered expression levels of several genes. It has been reported that the causal SNP in MERTK locus is represented by rs6726639, located within the binding sites of Transcriptional Repressor Factor (TF) which is able to bind to the A allele more strongly (12, 14). On the other hand, we suggested in our previous work that the down regulation of MERTK, observed in NAFLD patients with rs4374383 AA genotype, could be due to the association with rs6726639 AA genotype (11). In the same way, this genotype could influence the transcription of genes involved in fibrogenesis and in tumor suppression in CHC patients. Here, we evaluated the expression of MERTK and downstream signaling molecules in liver biopsies from CHC patients with AA (n=16) and GG (n=19) genotypes in the absence of HCC.

Secondly, in 12 selected liver samples genotyped for rs4374383 A>G and rs6726639 A>C SNPs of MERTK, we analyzed the expression of genes involved in fibrogenesis and HCC development. Depending on the polymorphic status of MERTK, we found a differential expression of Matrix Metallopeptidase 9 (MMP9), Matrix Metallopeptidase 7 (MMP7), Secreted Frizzled Related Protein 1 (SFRP1) and WNT gene family 11(WNT11) in the distinct groups of patients with CHC with or without HCC progression, thus confirming the role of MMPs and noncanonical WNT cascade in liver fibrogenesis and carcinogenesis.

## Materials and methods

### Patients

Selected tissue liver samples, available for 39 of the 349 CHC patients previously recruited at the Gastrointestinal and Hepatic Unit of the University of Palermo and genotyped for rs4374383 A> G SNP and for rs6726639 A> C SNP of MERTK were used (**Table 1**). Among them, 35 were analyzed in Real Time PCR to quantify the expression of MERTK and downstream pathways genes (**Tables 2**, **3**). Hepatic expression of genes involved in fibrogenesis or in tumor development/progression, listed in **Table 4**, was investigated in a subgroup of 9 patients chronically infected with HCV genotype-1b, monitored since 2013 in our hospital, and showing F3-F4 grade fibrosis (CHC). Among them, 2 developed HCC during the follow-up. The healthy subjects, used as control group, were constituted by two patients with histological diagnosis of NAFLD but without fibrosis and one patient without liver disease who underwent surgery for another pathology. The distribution of rs4374383 SNP and linked rs6726639 SNP genotypes was respectively: 7 AA/AA and 5 GG/CC (**Table 5**).

**Table 1:** Demographic, clinical, laboratory, and histological characteristics of CHC patients according to rs4374383 MERTK genotype.

	GG (20 pts)	AA (19 pts)	P value
Mean Age (years)	67.5	54.6	0.012
M/F	15-May	15-Apr	0.77
Esophageal varices	4 ()	3	0.85
Bioumoral data			
• AST (U/l)	90.7	48.2	0.08
• ALT (U/l)	114.4	57.4	0.07
• Bilirubin (mg(dl)	0.7	0.7	0.97
• Albumin (g/dl)	3.9	4	0.24
• INR	1.1	0.9	0.17
• Hb (g/dl)	14.4	14.8	0.66
• WBC (mmc)	6.38	6.505	0.84
• PLT (mmc)	184.941	155.437	0.16
• Creatinine (mg/dl)	0.7	0.8	0.93
Virological features			
viral load (mean)	2.275.333	1.776.750	0.56
HCV Genotypes			0.23
• 1a	0	2	
• 1b n. %	17 (85)	15 (79)	
• 2	1	1	
• 3	0	1	
• 4	0	0	
HBV	0	0	
HIV	0	0	
Liver stiffness by TE (Fibroscan)	14.9	10.1	0.04

**Table 2:** List of 24 target genes of RT2 Profiler PCR Array (QIAGEN).

24 target genes	
1. MERTK
2. PIK3CA
3. PIK3CB
4. PIK3CG
5. MAPK1
6. MAP2K
7. GRB2
8. STAT1
9. STAT5A
10. STAT5B
11. STAT6
12. BCL2
13. BCL2L1
14. BXL-XL
15. TLR4
16. TLR9
17. TRAF3
18. TRAF6
19. SOCS1
20. SOCS3
21. MYD88
22. FAS
23. FASLG
24. AKTIP

**Table 3:** Distribution of MERTK rs4374383 and rs6726639 SNPs in CHC patients selected for MERTK and downstream pathways panel.

SNP rs4374383 A> G of MERTK	SNP rs6726639 A> C of MERTK
AA	AA
AA	AA
AA	AA
GG	CC
GG	CC
GG	CC
GG	CC
AA	AA
GG	CC
AA	AA
AA	AA
AA	AA
GG	CC
GG	CC
GG	CC
AA	AA
GG	CC
AA	AA
AA	AA
AA	AA
AA	AC
AA	AA
AA	AA
AA	AA
AA	AA
GG	CC
GG	CC
GG	CC
GG	CC
GG	CC
GG	CC
GG	CC
GG	CC
GG	CC
GG	CA

**Table 4:** List of 88 genes target of Prime PCR card of epithelial-mesenchymal transition, stemness and metastagenesis panel.

88 target genes		
1. AXIN1	31. ITGB1	61. SFRP4
2. AXIN2	32. JAG1	62. SMAD2
3. BMP1	33. KRT14	63. SMAD4
4. BMP2	34. KISS1	64. SNAI1
5. BMP7	35. KISS1R	65. SNAI2
6. CCND1	36. MMP2	66. SNAI3
7. CD44	37. MMP3	67. SOX17
8. CDH1	38. MMP7	68. SRC
9. CDH2	39. MMP9	69. STAT3
10. COL1A1	40. KREMEN1	70. TGFB1
11. CDKN2A	41. KRT14	71. TGFB2
12. CTNNB1	42. LRP5	72. TGFB3
13. CXCL12	43. LRP6	73. TIMP1
14. CXCR4	44. MDM2	74. TIMP2
15. DKK1	45. MET	75. TIMP3
16. EGFR	46. MMP10	76. TIMP4
17. EPHB2	47. MMP11	77. TP53
18. ERBB2	48. MMP13	78. TWIST1
19. ERBB3	49. MYC	79. TSHR
20. ERBB4	50. NODAL	80. VIM
21. ESR1	51. NOTCH1	81. WNT11
22. FN1	52. OCLN	82. WNT3A
23. FZD7	53. PDGFRB	83. WNT5A
24. FGFR4	54. PTEN	84. WNT5B
25. FGFBP1	55. RAC1	85. WNT7A
26. GSK3B	56. RB1	86. WNT7B
27. HGF	57. RGS2	87. ZEB1
28. ILKAP	58. RHOA	88. ZEB2
29. ITGA5	59. SERPINE1	
30. ITGAV	60. SFRP1	

**Table 5:** Distribution of MERTK rs4374383 and rs6726639 SNPs in patients selected for epithelial-mesenchymal transition, stemness and metastagenesis panel.

Patient CHC	SNP RS4374383 A>G of MERTK	SNP rs6726639 A>C of MERTK
CHC	AA	AA
CHC	GG	CC
HCC	AA	AA
CHC	AA	AA
CHC	AA	AA
CHC	AA	AA
CHC	GG	CC
CHC	GG	CC
HCC	AA	AA
HEALTHY	GG	CC
HEALTHY	GG	CC
HEALTHY	AA	AA

Patients with alcohol intake> 20g/per day, autoimmune hepatitis, hereditary hemochromatosis, α1-antitrypsin deficiency, and severe obesity (BMI> 40Kg/m2) were excluded.

The study was conducted in accordance with the principles of the Declaration of Helsinki and with local and national laws. The approval was obtained by the Comitato Etico Palermo 1 ID 2014 - AOUP “Paolo Giaccone” in Palermo.

### DNA isolation and genotyping

DNA was purified using the QIAmp blood Mini Kit (Qiagen, Mainz,Germany) and DNA samples were quantified using spectrophotometric determination. Genotyping for MERTK (rs4374383 A>G SNP and rs6726639 A>C SNP) was carried out using the TaqMan SNP genotyping allelic discrimination method (Applied Biosystems, Foster City, CA, USA). Genotyping assays were commercially available. Genotypes were called using Sequence Detection Software (SDS v.2.3) (Applied Biosystems, USA). Genotyping was conducted in a blinded fashion relative to patient characteristics.

### RNA isolation and real-time PCR microarray

Total RNA was purified from liver biopsies by miRNeasy Micro Kit (Qiagen) and quantified using NanoDrop ™ 1000 Spectrophotometer (ThermoFisher Scientific). 1 µg of RNA retro-transcribed using the iScript ™ gDNA Clear cDNA Synthesis Kit according to manufacturer’s recommendations (Bio-Rad, CA, USA). Relative expression level of target genes was evaluated by real-time PCR using custom RT2 Profiler PCR Array (Qiagen) or Prime PCR Custom Panel as recommended by manufacturer (Bio-Rad, CA, USA). Quantitative Real Time PCR was performed using 96 wells plates (Qiagen, 24 target genes listed in **Table 3**) or 384 well-plate pre-designed Prime PCR card of epithelium-to-mesenchyme transition stemness and metastagenesis panel (Bio-Rad, CA, USA, 88 target genes listed in **Table 4**). All the plates contained primers for genomic DNA detection (gDNA), positive PCR control (PCR), RNA Quality Assay (RQ1 and RQ2), Reverse Transcription Control (RT) and 2 housekeeping genes: TATA-box binding protein (TBP), and hypoxanthine phospho-ribosyl-transferase 1 (HPRT1). Data were expressed as fold change using 2 ^− ΔΔCt^ method referred to NAFLD F0-F1 patient as control sample. Differences among experimental groups were analyzed by Student t test and used for comparison with PRIME PCR analysis software (Bio-Rad) or data analysis software is available at http://www.qiagen.com/it/shop/genes-and-pathways/data-analysis-center-overview-page/.

### Statistical methods

Based on the relative quantification method, the amount of target, normalized to the endogenous reference HPRT1 and relative with respect to a control sample (NAFLD f0-f1 patient), was computed. A list of gene expression, for which RQ is statistically significant when analyzed among the experimental groups has been identified. The mean and the standard deviation was identified for relative gene expressions inside experimental groups of subjects with CHC and respectively AA or GG genotype, with NAFLD and AA or GG genotype, and without liver disease and GG genotype for rs rs4374383 SNP of MERTK; explorative analyses were carried out to verify conditions on distributions and similar population variability. The student’s t-test for independent experiments was performed for testing differences in fold expression of genes between the experimental groups; the Bonferroni correction for multiple hypothesis testing was applied to t-test and P-value < 0.05 was statistically significant. Statistical analysis was performed using R Statistical Software version 3.6.3 (15).

## Results

### MERTK liver expression and signaling pathways downstream panel

We evaluated liver expression of MERTK and downstream signaling pathways in liver biopsies from CHC patients with AA (n = 16) and GG (n = 19) genotypes in the absence of HCC.

**Figure 1:**
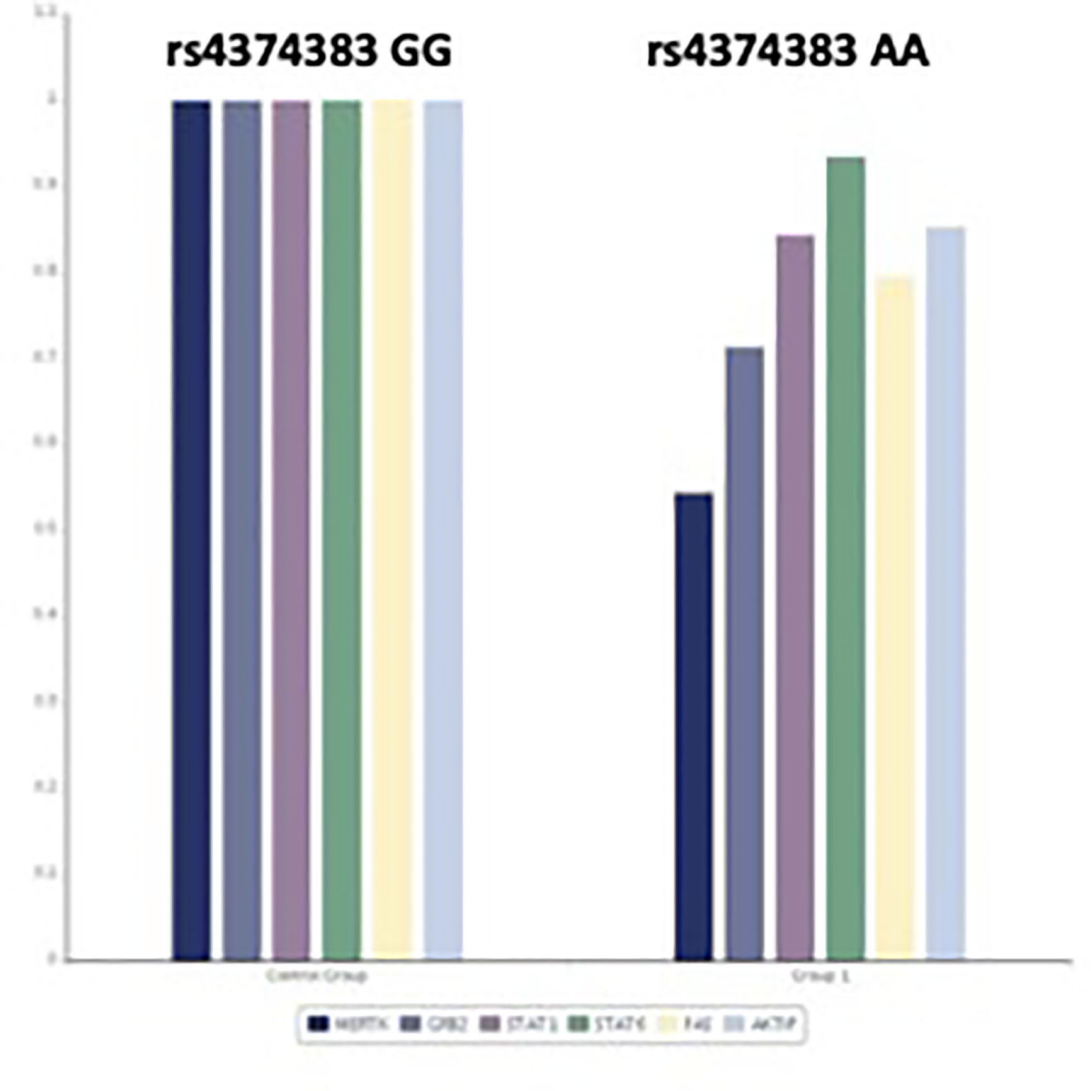
Differential gene expression in CHC patients with GG MERTK genotype (N = 19) and AA genotype (N = 16). P-VALUE: MERTK 0.01; GRB2 0.10; STAT1 0.36; STAT6 0.2; FAS 0.07; AKTIP 0.03.

Interestingly, we found that, although AA homozygosity was associated with lower MERTK expression (1.85 folds), the phosphatidylinositol-3-kinase (PI3K) was expressed in the same way, while other molecules downstream were downregulated in AA (**Figure 1**).

### Gene expression of epithelium-to-mesenchyme transition stemness and metastagenesis panel

Gene expression analyses were performed in a subset of 12 patients, listed in **Table 4**, with different MERTK genotypes for whom frozen liver biopsies were available (9 with CHC, 2 with NAFLD and 1 without liver disease). In order to test for conditional independence of up/down regulated results and different groups with respect to each gene, the Mantel-Haenszel X-squared test (MH) was implemented on the correspondent frequency tables, after having stratified by genes (Cochran-Mantel-Haenszel M^2 = 106.62, df = 12, p-value <0.001). Using an RT Profiler PCR microarray approach, a list of 4 genes, MMP7, MMP9, SFRP1 and WNT11, among the 54 analyzed, showed evidence of differential expression between the experimental groups CHC (AA genotype), CHC (GG genotype), HCC (AA genotype). The magnitude of these differences could be reasonably estimated and tested even with small sample sizes assuming standard hypothesis. The T-test detected statistically significant differences in fold expression of genes MMP9, MMP7, SFRP1 and WNT11. Evidence for differential expression was evaluated using statistical significance with p-values <0.05.

**Figure 2:**
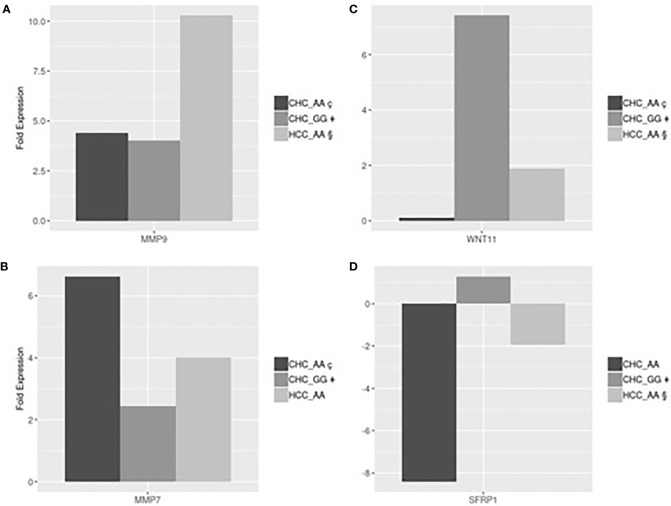
**(A)** HCC-AA vs CHC-AA and CHC-GG: statistically significant mean fold changes for MMP9 gene. PMMP9_§ç=0.045, PMMP9_§‡=0.04; **(B)** CHC-AA vs CHC-GG: statistically significant mean fold changes for MMP7 gene. PMMP7_ ç‡=0.01; **(C)** CHC-GG vs HCC-AA and vs CHC-AA: statistically significant mean fold changes for WNT11 gene. PWNT11_§‡=0.04; PWNT11_ç‡=0.03; **(D)** HCC-AA vs CHC-GG: statistically significant mean fold changes for SFRPI gene. PSFRPI §‡<0.01.

MMP9 was more strongly up-regulated in HCC-AA (mean 10.3-fold increase) than in either CHC-AA (mean 4.41-fold increase) and CHC-GG groups (mean 4.02-fold increase). In both comparisons differences turn out to be significant with p-values respectively 0.045 and 0.04 (**Figure 2A**). The mean fold expression of HCC-AA group is 2.33 times higher than CHC-AA and 2.55 times higher than CHC-GG mean. MMP7 is up regulated in CHC-AA group, and its mean fold expression (6.63) is statistically different with p-value <0.01 and 2.7 times higher when compared with mean fold expression of CHC-GG group (2.43) (**Figure 2B**). For WNT11 the mean fold change difference is both statistically significant when CHC-GG group (mean= 7.42) is compared with CHC-AA (mean=0.11 and p value= 0.01) and with HCC-AA (mean=1.88 and p value=0.03). Mean expression in CHC-GG is 5.8 higher than in CHC-AA and 3.9 times higher than HCC-AA (**Figure 2C**). For SFRP1, the mean fold expression of CHC-GG and HCC-AA groups (1.29 and 1.94, with relative normalized expression in CHC-GG 2.5 times higher than HCC-AA) are statistically different (t-test=8.63, p value <0.01**)** (**Figure 2D**).

## Discussion

MerTK is expressed in human HSCs and acts directly on these cells by promoting their activation, supporting their survival, increasing their migration, and inducing the expression of profibrogenic genes, such as procollagen type I. We have previously showed that the AA genotypes of MERTK, associated with lower MerTK hepatic expression, is protective against severe fibrosis in NAFLD patients and is able to condition the progression of this chronic liver disease. By contrast, the GG genotype has been associated with fibrotic phenotype in NAFLD, showing a potential direct profibrotic action of MerTK (11).

MerTK, one of the three RTKs of TAM family, is expressed predominantly in M2 cells and acts inhibiting the inflammatory responses and controlling the tissue tolerance (16; 17). It plays a functional role in the clearance of apoptotic cells and this function is restricted to a subset of M2 macrophages, named M2c, that are characterized by secretion of IL-10 and up-regulation of MerTK (18). In fact, M2 differentiation is a key process that regulates inflammation and fibrosis (19). In chronic disease such as NAFLD, these macrophage populations, through MerTK play a crucial role to induce the HSCs trans differentiation in myofibroblasts promoting fibrogenesis. Gas6, together with the other ligands, binds to MerTK and induces its dimerization and autophosphorylation, so activating the downstream signaling (Linger et Al.,2008; 20). In addition, MerTK suppresses TLR signaling and down-regulates the production of pro-inflammatory cytokines through the phosphorylation of STAT1 which promotes the induction of SOCS-1 and SOCS-3 (21, 22). Normally, MerTK releases factors involved in tissue remodeling, immune responses suppression and tumor-promoting (23, 24).

Our evidence suggests that detection of the MERTK polymorphic status rs4374383, along with rs6726639, should warn in favor of more stringent monitoring to early detect disease progression and cancer development in patients with CHC and CHB and genotype AA/AG or progression to cirrhosis and its complications in patients with NAFLD and genotype GG/AG (9–11).

However, how MerTK can condition the progression of liver disease remains unknown. The aim of our work was to investigate the functional mechanism that links MerTK and progression of liver disease in CHC patients especially in neoplastic direction. We have focused our attention on the polymorphic status of MERTK rs6726639 because the A allele is able to differentially bind to transcription factors, thus conditioning MERTK expression and affecting downstream pathways (12). In this regard, in our CHC patients, we found that AA genotype is associated with lower expression of MERTK (1.8 folds) and with consequent lower expression of other downstream molecules compared to GG genotype (**Figure 1**).

In the same way, the AA genotype could negatively affect the transcription of genes involved in fibrogenesis and in tumor suppression. For this purpose, we have selected a subset of patients with CHC, for whom liver samples were available, with or without neoplastic progression in respect to the polymorphic MERTK status. The main finding of the present work was the differential expression of

MMP9, MMP7, SFRP1 and WNT11 in the different groups.

MMPs play a key role in several processes including epithelial-mesenchymal transition and are involved in fibroproliferative process and HCC development and progression during HCV infection (25, 26). Our results are in line with these suggestions showing, in **Figure 2A**, that MMP9 was upregulated in HCC-AA compared to both CHC-AA and CHC-GG groups, confirming the role of MMP9 in hepatocarcinogenesis. On the other hand, MMP7 was found upregulated in CHC-AA group compared with CHC-GG group (**Figure 2B**), confirming the role of AA genotype in conditioning the expression of genes involved in neoplastic progression.

Another pathway that was found differentially expressed was the WNT11/SRP1 pathway.

It has been demonstrated that WNT pathway is regulator of hepatic progenitor cells differentiation: canonical WNT signaling promotes the differentiation into hepatocytes, while noncanonical WNT signaling promotes the differentiation into myofibroblast and the consequent progression of liver fibrosis (27). Among the members of noncanonical cascade, WNT11 was reported to have a role in hepatic oncogenesis. In particular, the expression levels of WNT11 were significantly downregulated in human HCC, thus suggesting a tumor-suppressing role for WNT11 (28). Our results are totally in accord with this suggestion being WNT11 expression in CHC-GG 5.8 higher than in CHC-AA and 3.9 times higher than in HCC-AA (**Figure 2C**). On the other hand, we found that the expression of SFRP, which is a negative modulator of WNT canonical pathway (29), is higher in CHC-GG than in HCC-AA (**Figure 2D**). Moreover, we suggest that the observed differential expression of WNT11 and SFRP1 which work together to the activation of noncanonical WNT pathway, is related to MERTK polymorphic status.

We are aware that a major criticism of the present work is represented by the small size of human liver samples, due to the lack of a liver biopsy approach in the era of direct-acting antivirals. Nevertheless, due to the magnitude of differential gene expression, our hypotheses appear reasonable.

Certainly, our present data represent a first step, and, in the next future, we are planning to implement the sample size, including tissues from liver diseases with different etiology (viral and metabolic), focusing our attention on the pathways suggested by this preliminary work.

From the clinical perspective, our study strongly suggests that the rs4374383/rs6726639 SNPs of MERTK may represent a useful genetic biomarker to identify those patients with chronic liver diseases who are at high risk of progression and therefore deserve a more rigorous follow-up for HCC surveillance. Unraveling the mechanisms involved in hepatocarcinogenesis have intrinsic translational relevance to identify key pathways for clinical approach.

## Data availability statement

The datasets presented in this study can be found in online repositories. The names of the repository/repositories and accession number(s) can be found below:

https://www.ncbi.nlm.nih.gov/, NM_006343.2

https://www.ncbi.nlm.nih.gov/, NM_006343.3

https://www.ncbi.nlm.nih.gov/, NM_004994.3

https://www.ncbi.nlm.nih.gov/, NM_003012.5

https://www.ncbi.nlm.nih.gov/, NM_004626.3.

## Ethics statement

The study was conducted in accordance with the principles of the Declaration of Helsinki and with local and national laws. The approval was obtained by the Comitato Etico Palermo 1 ID 2014 - AOUP “Paolo Giaccone” of Palermo. The patients/participants provided their written informed consent to participate in this study.

## Author contributions

Conceptualization: RP, SG, and AC. Methodology: RZ, CM, GL, and MG. Software: FS and MR. Validation: FS. Formal analysis: FS and MR. Investigation: RP, SG, LM, VC, VM, and SP. Data curation: RP and FS. Writing: RP and SG. Original draft preparation, review and editing: RP, SG, and AC. Supervision, SG and AC. All authors have read and agreed to the published version of the manuscript. All authors contributed to the article and approved the submitted version.

## Conflict of interest

The authors declare that the research was conducted in the absence of any commercial or financial relationships that could be construed as a potential conflict of interest.

## Publisher’s note

All claims expressed in this article are solely those of the authors and do not necessarily represent those of their affiliated organizations, or those of the publisher, the editors and the reviewers. Any product that may be evaluated in this article, or claim that may be made by its manufacturer, is not guaranteed or endorsed by the publisher.
